# Publisher Correction: Female mice lacking Ftx lncRNA exhibit impaired X-chromosome inactivation and a microphthalmia-like phenotype

**DOI:** 10.1038/s41467-018-07100-5

**Published:** 2018-10-31

**Authors:** Yusuke Hosoi, Miki Soma, Hirosuke Shiura, Takashi Sado, Hidetoshi Hasuwa, Kuniya Abe, Takashi Kohda, Fumitoshi Ishino, Shin Kobayashi

**Affiliations:** 1 Department of Epigenetics, Medical Research Institute, Tokyo Medical and Dental University (TMDU), 1-5-45 Yushima, Bunkyo-ku, Tokyo 113-8510, Japan; 2 Molecular Profiling Research Center for Drug Discovery, National Institute of Advanced Industrial Science and Technology, 2-4-7 Aomi, Koutou-ku, Tokyo 135-0064, Japan; 3 Technology and Development Team for Mammalian Genome Dynamics, RIKEN BioResource Research Center, 3-1-1 Koyadai, Tsukuba, Ibaraki 305-0074 Japan; 4 Department of Bioscience, Graduate School of Agriculture, Kindai University, 3327-204, Nakamachi, Nara 631-8505 Japan; 5 Research Institute for Microbial Diseases, Osaka University, Yamadaoka 3-1, Suita, Osaka 565-0871 Japan; 6 Faculty of Life and Environmental Sciences, University of Yamanashi, 4-4-37 Takeda, Kofu, Yamanashi 400-8510 Japan; 7 Present Address: Department of Molecular Biology, Keio University School of Medicine, Tokyo 160-8582, Japan

Correction to: *Nature. Communications.*; 10.1038/s41467-018-06327-6, published online 20 Sep 2018.

In the original HTML version of this Article, the affiliation details for Hirosuke Shiura were incorrectly given as: ‘Department of Epigenetics, Medical Research Institute, Tokyo Medical and Dental University (TMDU), 1-5-45 Yushima, Bunkyo-ku, Tokyo, 113-8510, Japan; Technology and Development Team for Mammalian Genome Dynamics, RIKEN BioResource Research Center, 3-1-1 Koyadai, Tsukuba, Ibaraki, 305-0074, Japan; Present address: Faculty of Life and Environmental Sciences, University of Yamanashi, 4-4-37 Takeda, Kofu, Yamanashi, 400-8510, Japan’. The correct affiliations are: ‘Department of Epigenetics, Medical Research Institute, Tokyo Medical and Dental University (TMDU), 1-5-45 Yushima, Bunkyo-ku, Tokyo 113-8510, Japan; Technology and Development Team for Mammalian Genome Dynamics, RIKEN BioResource Research Center, 3-1-1 Koyadai, Tsukuba, Ibaraki 305-0074, Japan; Faculty of Life and Environmental Sciences, University of Yamanashi, 4-4-37 Takeda, Kofu, Yamanashi 400-8510, Japan’.

Also, in the original HTML version of this Article, the affiliation details for Hidetoshi Hasuwa were incorrectly given as: ‘Research Institute for Microbial Diseases, Osaka University, Yamadaoka 3-1, Suita, Osaka, 565-0871, Japan’. The correct affiliations are: ‘Research Institute for Microbial Diseases, Osaka University, Yamadaoka 3-1, Suita, Osaka, 565-0871, Japan; Present address: Department of Molecular Biology, Keio University School of Medicine, Tokyo, 160-8582, Japan’.

Additionally, in the original HTML version of this Article, the affiliation details for Takashi Kohda were incorrectly given as: ‘Department of Epigenetics, Medical Research Institute, Tokyo Medical and Dental University (TMDU), 1-5-45 Yushima, Bunkyo-ku, Tokyo, 113-8510, Japan; Present address: Department of Molecular Biology, Keio University School of Medicine, Tokyo, 160-8582, Japan’. The correct affiliations are: ‘Department of Epigenetics, Medical Research Institute, Tokyo Medical and Dental University (TMDU), 1-5-45 Yushima, Bunkyo-ku, Tokyo, 113-8510, Japan; Faculty of Life and Environmental Sciences, University of Yamanashi, 4-4-37 Takeda, Kofu, Yamanashi 400-8510, Japan’.

These have now been corrected in the HTML version of the Article.

