# Supplementary material for: Female mice lacking Ftx lncRNA exhibit impaired X-chromosome inactivation and a microphthalmia-like phenotype
Source: Nat Commun. 2018 Sep 20;9:3829. doi: 10.1038/s41467-018-06327-6 (PMC6148026; doi:10.1038/s41467-018-06327-6)
Supplement: Supplementary file 1 — Supplementary Information [file 41467_2018_6327_MOESM1_ESM.pdf]

Supplemental Information

Female mice lacking *Ftx* lncRNA exhibit impaired X-chromosome inactivation and a microphthalmia-like phenotype

Yusuke Hosoi, Miki Soma, Hirosuke Shiura, Takashi Sado, Hidetoshi Hasuwa, Kuniya Abe , Takashi Kohda, Fumitoshi Ishino, Shin Kobayashi

Supplementary Figure 1–12
Supplementary Table 1 – 7
Supplementary Data 1 and 2
References

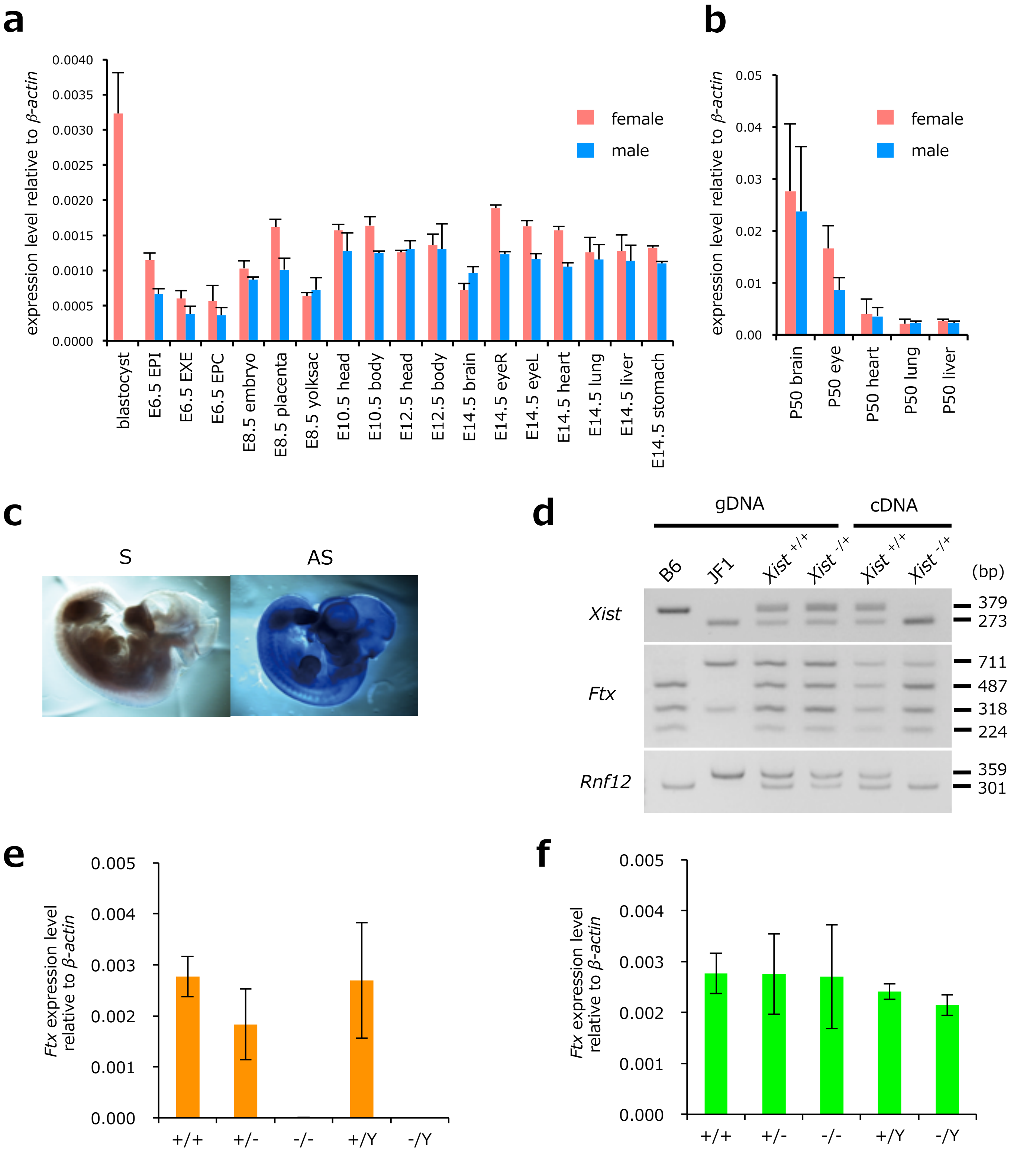

Supplementary Figure 1. Expression of *Ftx* lncRNA in developing embryos and adult tissues.
(**a**) RT–qPCR quantification of *Ftx* in developing male and female embryos (*n* = 3). *β-actin*, beta-actin. Error bars, s.d. (**b**) RT–qPCR quantification of *Ftx* in adult male and female tissues (*n* = 3). P, postnatal day. Error bars, s.d. (**c**) Whole-mount *in situ* hybridization of *Ftx* in an E11.5 embryo. Ubiquitous expression of *Ftx* was detected. AS, antisense strand probe; S, sense strand probe. (**d**) Allelic expression analysis of *Xist* and *Ftx* in *Xist*-deficient MEF cells (–B6/+JF1). *Ftx* escaped XCI and was biallelically expressed from both active and inactive X chromosomes. *Rnf12* subject to random XCI was used as a control. (**e**) *Ftx* expression was not detected in *Ftx*$^{-/-}$ mice. The expression levels of *Ftx* were quantified by RT–qPCR in adult kidneys (*n* = 3 in each group) of wild-type females (+/+), heterozygous females (+/–), homozygous females (–/–), wild-type males (+/Y) and hemizygous males (–/Y). Error bars, s.d. (**f**) RT–qPCR quantification of *Ftx* in *miRNA374/421*-DKO mice. The expression levels of *Ftx* were quantified by RT–qPCR in adult kidneys (*n* = 3 in each group) of WT females (+/+), heterozygous females (+/–), homozygous females (–/–), WT males (+/Y) and hemizygous males (–/Y). Error bars, s.d.

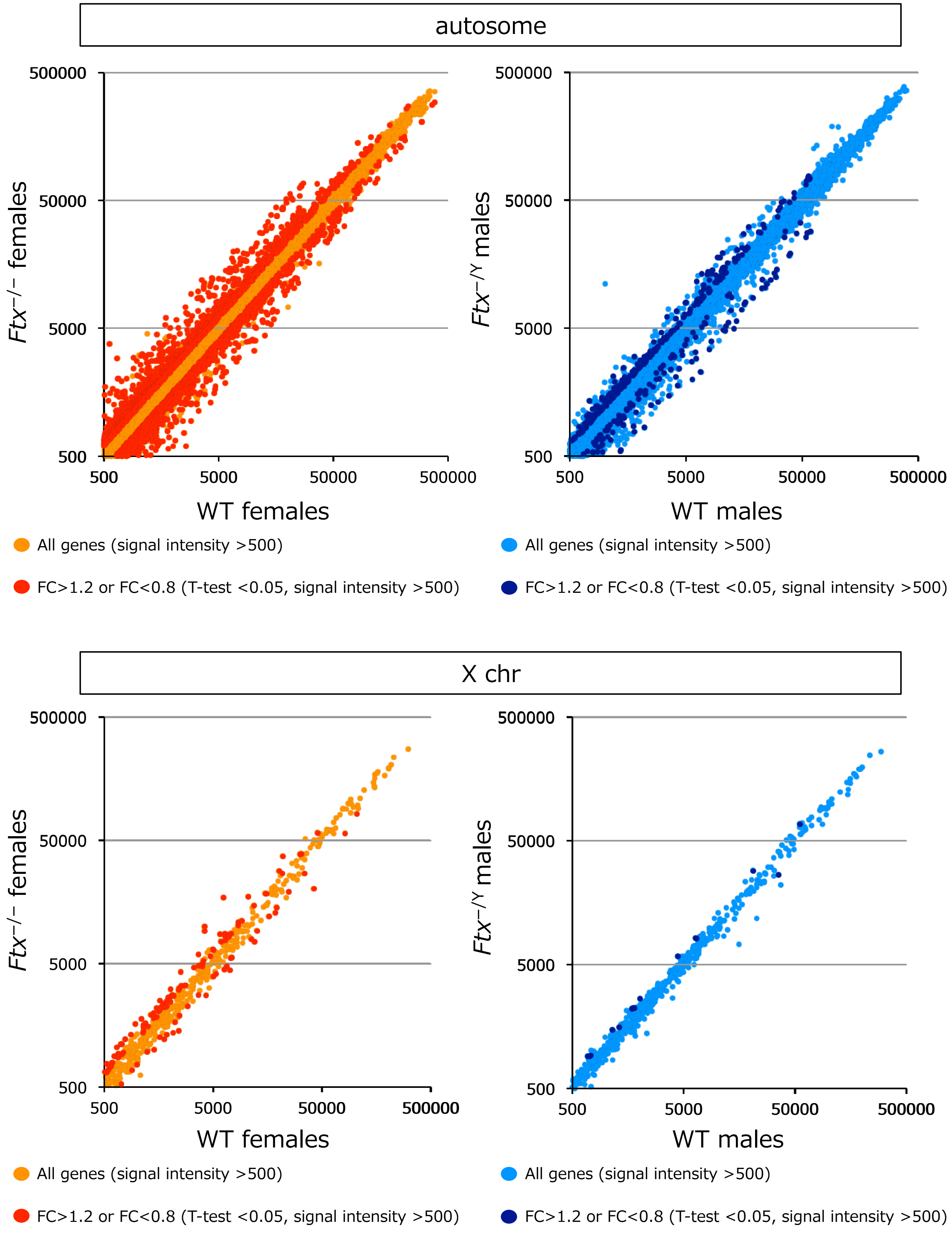

Supplementary Figure 2. Microarray expression profiling.
Scatter plot comparing wild-type and E13.5 *Ftx* mutant samples (upper, autosomal genes; lower, X-linked genes). All genes selected for this analysis showed a relative signal intensity of > 500 in female eyes in Agilent microarrays.

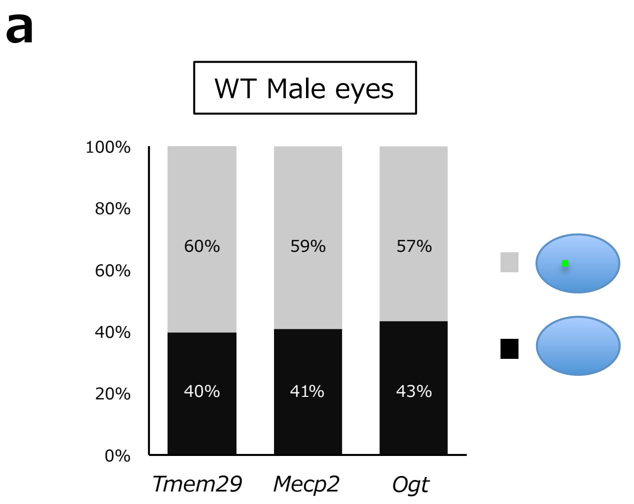

n=3	*Tmem29*	*Mecp2*	*Ogt*
1	45	45	36
2	32	43	36
3	33	42	46
Total nuclei	110	130	118

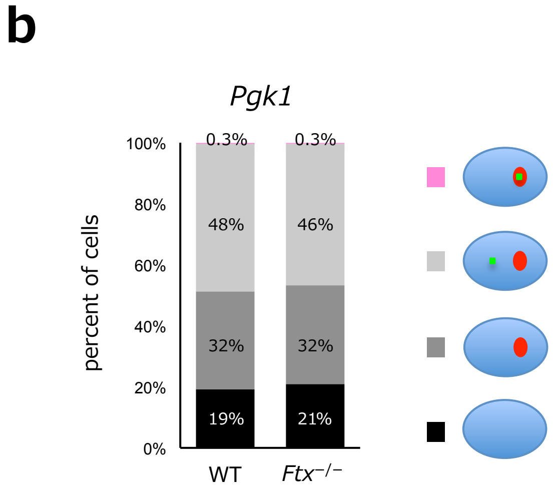

	Pgk1	
n=3	WT	*Ftx*$^{-/-}$
1	117	101
2	98	96
3	112	114
Total nuclei	327	311

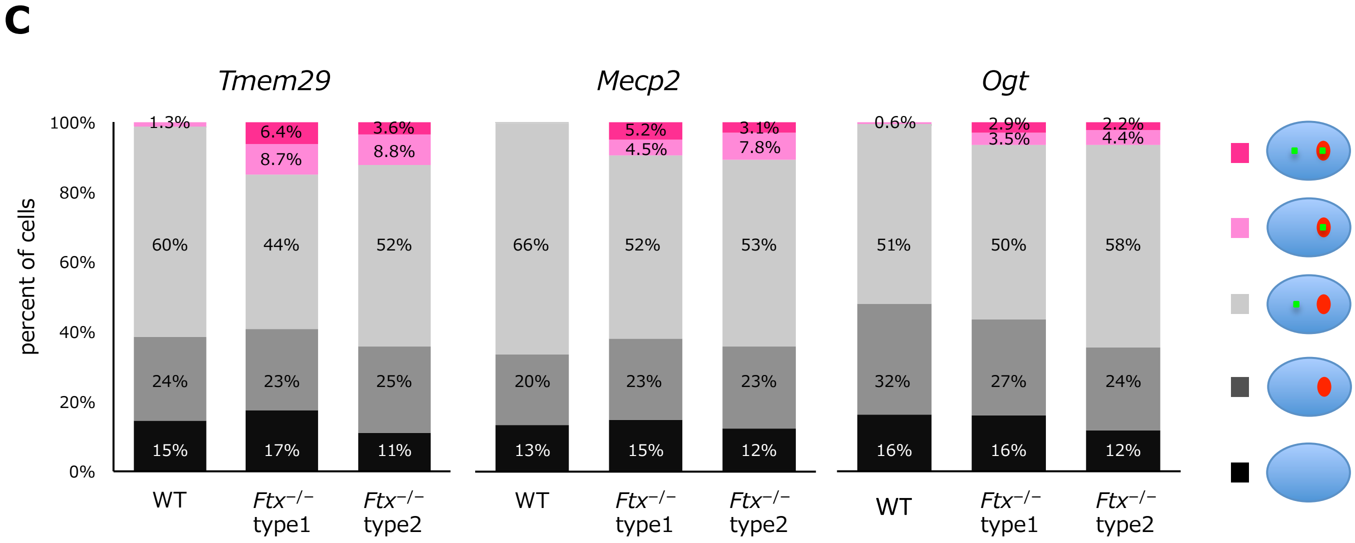

	Tmem29			*Mecp2*			*Ogt*		
n=3	WT	*Ftx*$^{-/-}$ type1	*Ftx*$^{-/-}$ type2	WT	*Ftx*$^{-/-}$ type1	*Ftx*$^{-/-}$ type2	WT	*Ftx*$^{-/-}$ type1	*Ftx*$^{-/-}$ type2
1	91	108	43	127	112	42	115	119	46
2	128	93	49	120	96	45	93	119	43
3	91	110	45	81	102	42	104	101	46
Total nuclei	310	311	137	328	310	129	312	339	135

＊ : P<0.01

＊＊ : P<0.001

Supplementary Figure 3. RNA–FISH analysis of eyes isolated from embryos at E13.5.
(**a**) Summary of *Tmem29, Mecp2* and *Ogt* expressions in combination with *Xist in* male eyes. Approximately 40 nuclei in each eye were counted from three independent experiments and classified based on the patterns of *Xist* clouds and nascent X-linked gene transcripts. *Xist* signals were not observed in male eyes. (b) Summary of *Pgk1*: a non-upregulated gene in the microarray analysis. The prevalence of nuclei showing respective expression patterns of *Xist* clouds and nascent X-linked gene transcripts are depicted. Total cell numbers analysed in each embryo are shown in the table. No statistically significant differences between WT and *Ftx*$^{-/-}$ mice were observed for the non-upregulated gene *Pgk1*. (c) Summary of RNA–FISH analysis of the *Ftx*$^{-/-}$ type 2 eyes compared with WT, *Ftx*$^{-/-}$ type 1 eyes. RNA–FISH detected X-linked genes: *Tmem29, Mecp2,* and *Ogt*, in combination with *Xist*. Statistically significant differences between *Ftx*$^{-/-}$ type 1 and type 2 mice were not observed.

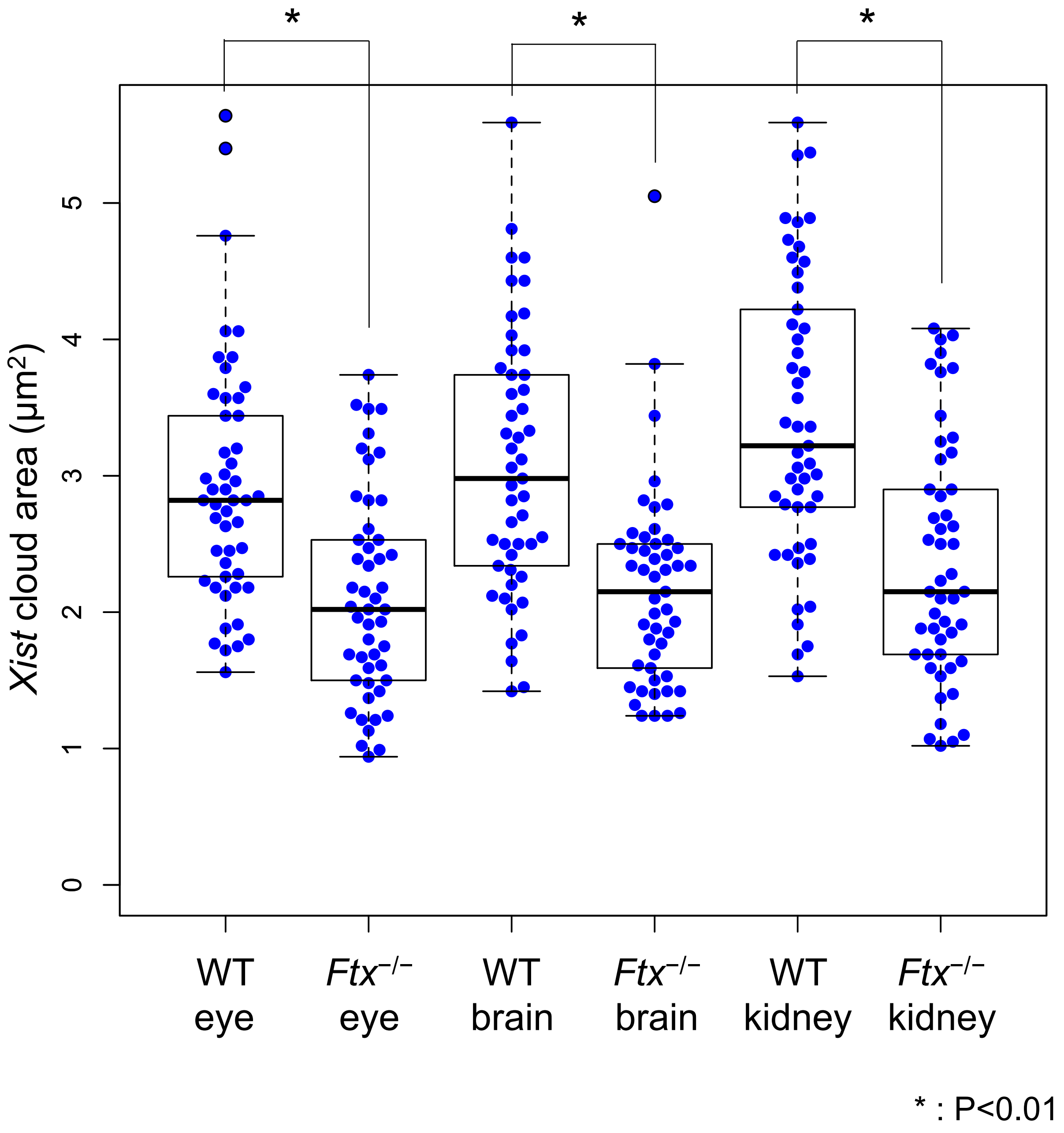

Supplementary Figure 4. The small *Xist* cloud detected in *Ftx*–/– embryos at E13.5.
The size of the *Xist* cloud area in RNA–FISH was measured using Image J software; 50 nuclei were measured for each sample and the mean cloud area was significantly different between the *Ftx*–/– and WT embryos (two-tailed, unequal variance *t*-test). The box represents the median, 25th and 75th percentiles; whiskers indicate the maximum and minimum values.

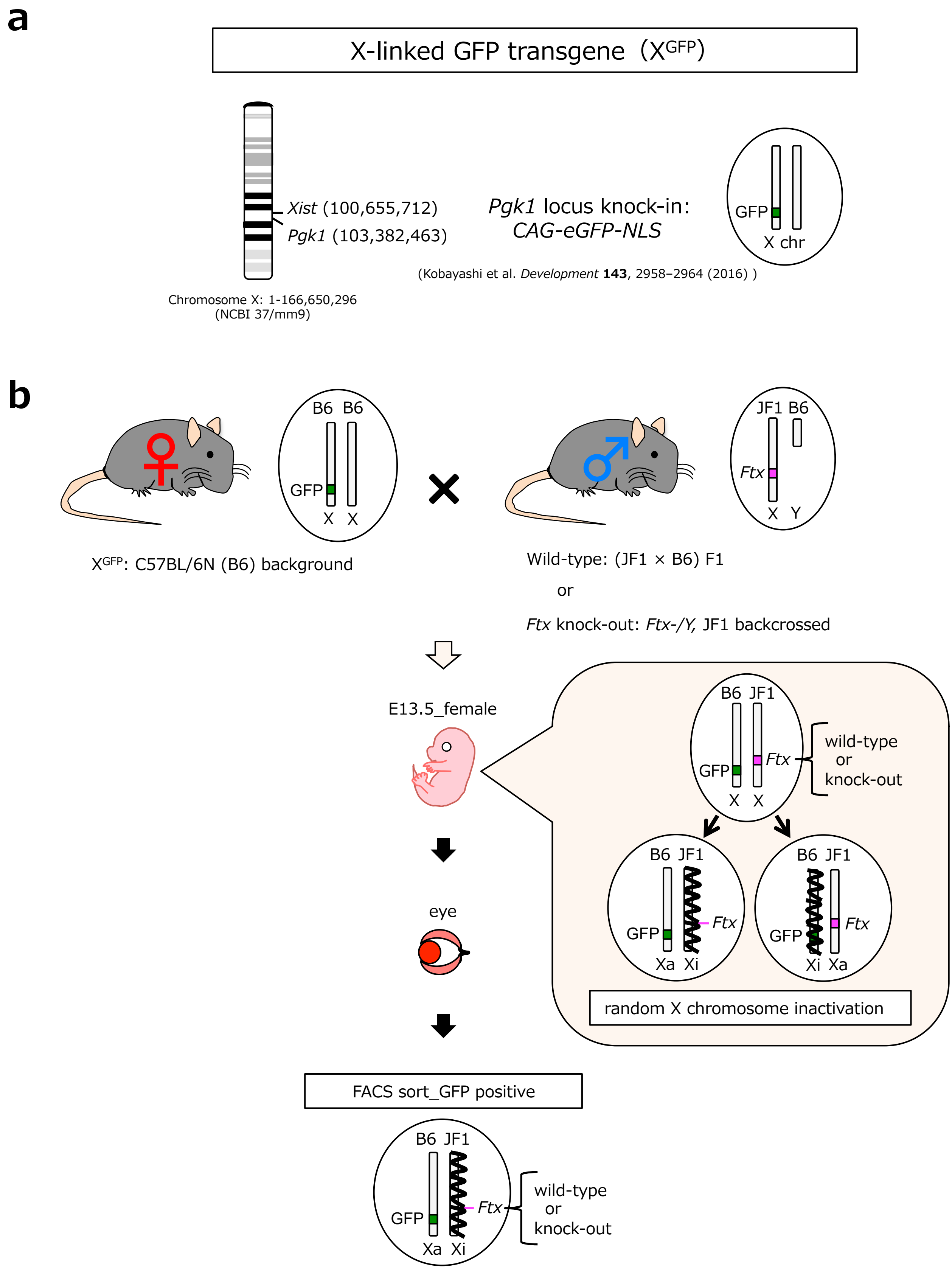

Supplementary Figure 5. Allelic expression analysis following random XCI at E13.5 eyes.
(**a**) The gene encoding the reporter protein eGFP was inserted into the *Pgk1* locus on X chromosomes (X^{GFP} mouse). (**b**) Scheme of the allelic expression analysis to confirm the impairment of random XCI in heterozygous $Ftx^{+/-}$ eyes. X^{GFP} mice with a *Mus musculus musculus* (C57BL/6N: B6) background and WT or Ftx^{-}/Y mutant mice with a *Mus musculus molossinus* (JF1) background were crossed and F1 embryos were recovered for RFLP analysis.

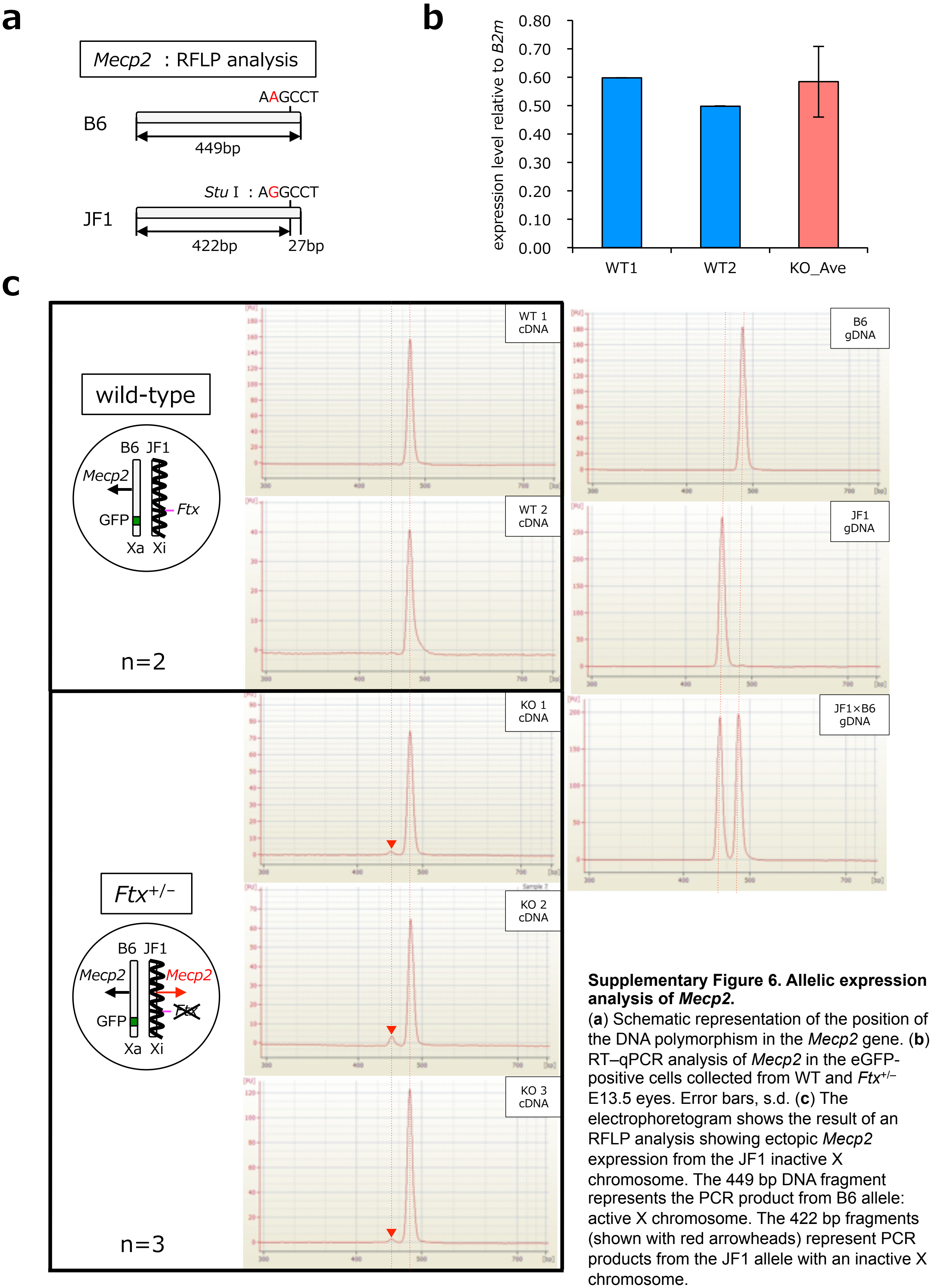

Supplementary Figure 6. Allelic expression analysis of *Mecp2*.
(**a**) Schematic representation of the position of the DNA polymorphism in the *Mecp2* gene. (**b**) RT–qPCR analysis of *Mecp2* in the eGFP-positive cells collected from WT and *Ftx*$^{+/-}$ E13.5 eyes. Error bars, s.d. (**c**) The electrophoretogram shows the result of an RFLP analysis showing ectopic *Mecp2* expression from the JF1 inactive X chromosome. The 449 bp DNA fragment represents the PCR product from B6 allele: active X chromosome. The 422 bp fragments (shown with red arrowheads) represent PCR products from the JF1 allele with an inactive X chromosome.

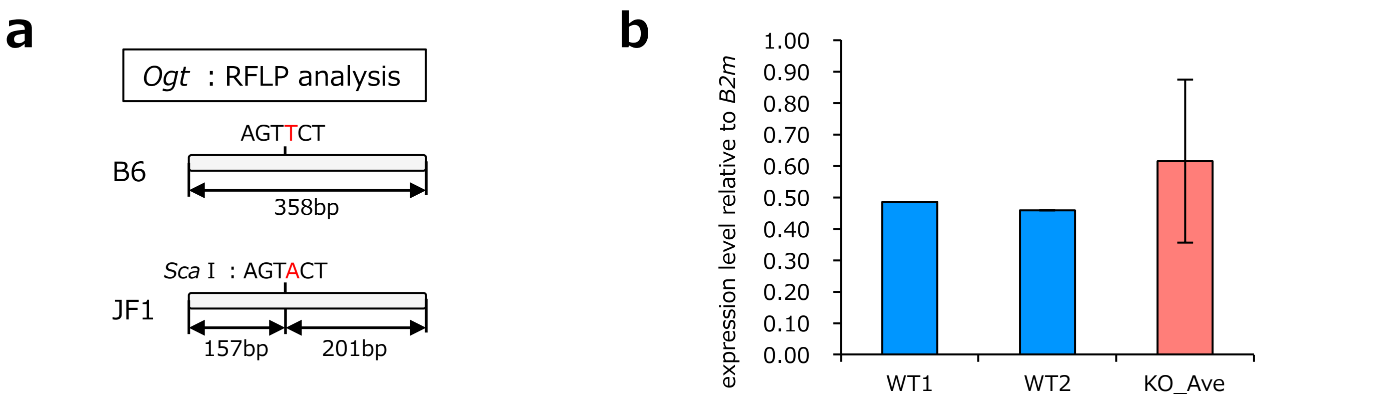

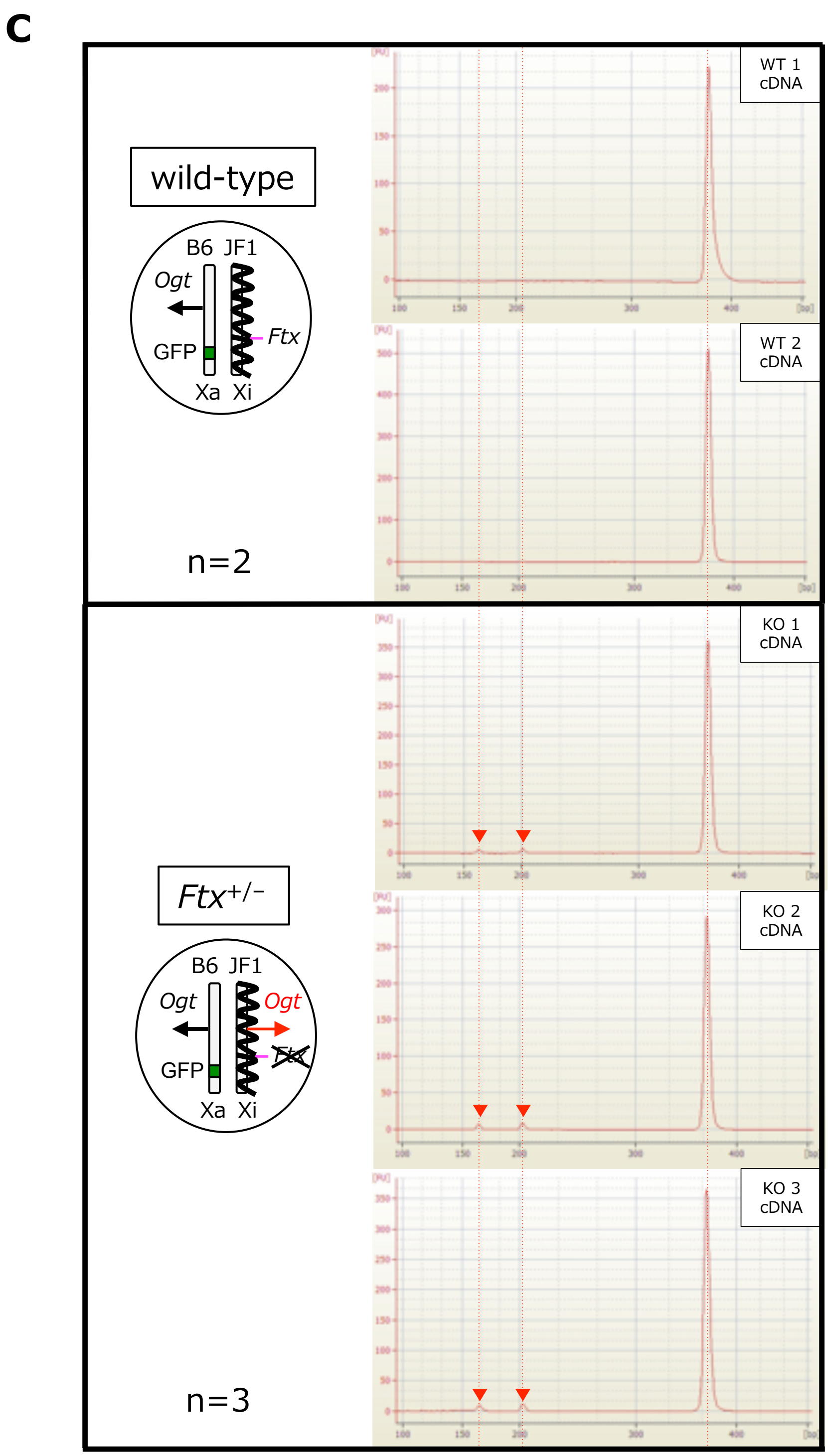

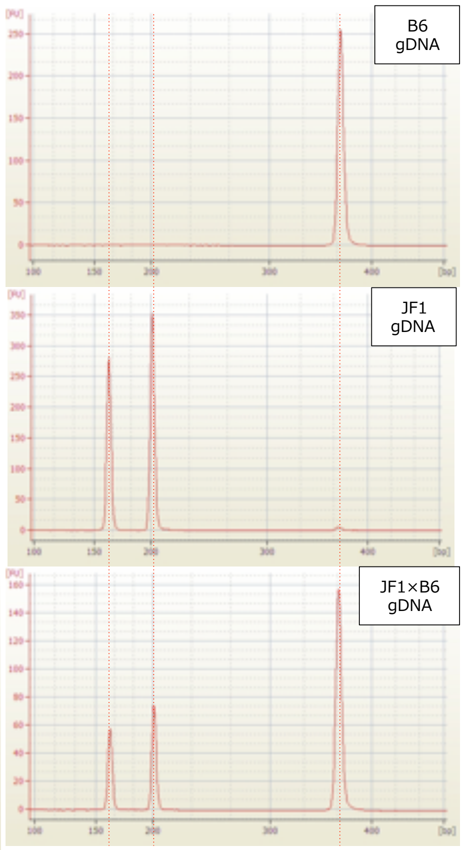

Supplementary Figure 7. Allelic expression analysis of *Ogt*.
(**a**) Schematic representation of the position of the DNA polymorphism in the *Ogt* gene. (**b**) RT–qPCR analysis of *Ogt* in the eGFP-positive cells collected from WT and *Ftx*$^{+/-}$ E13.5 eyes. Error bars, s.d. (**c**) The electrophoretogram shows the result of a RFLP analysis showing ectopic *Ogt* expression. The 358 bp DNA fragment represents the PCR product from B6 allele: the active X chromosome. The 157 bp and 201 bp fragments (shown with red arrowheads) represent the PCR products from JF1 alleles with an inactive X chromosome.

a

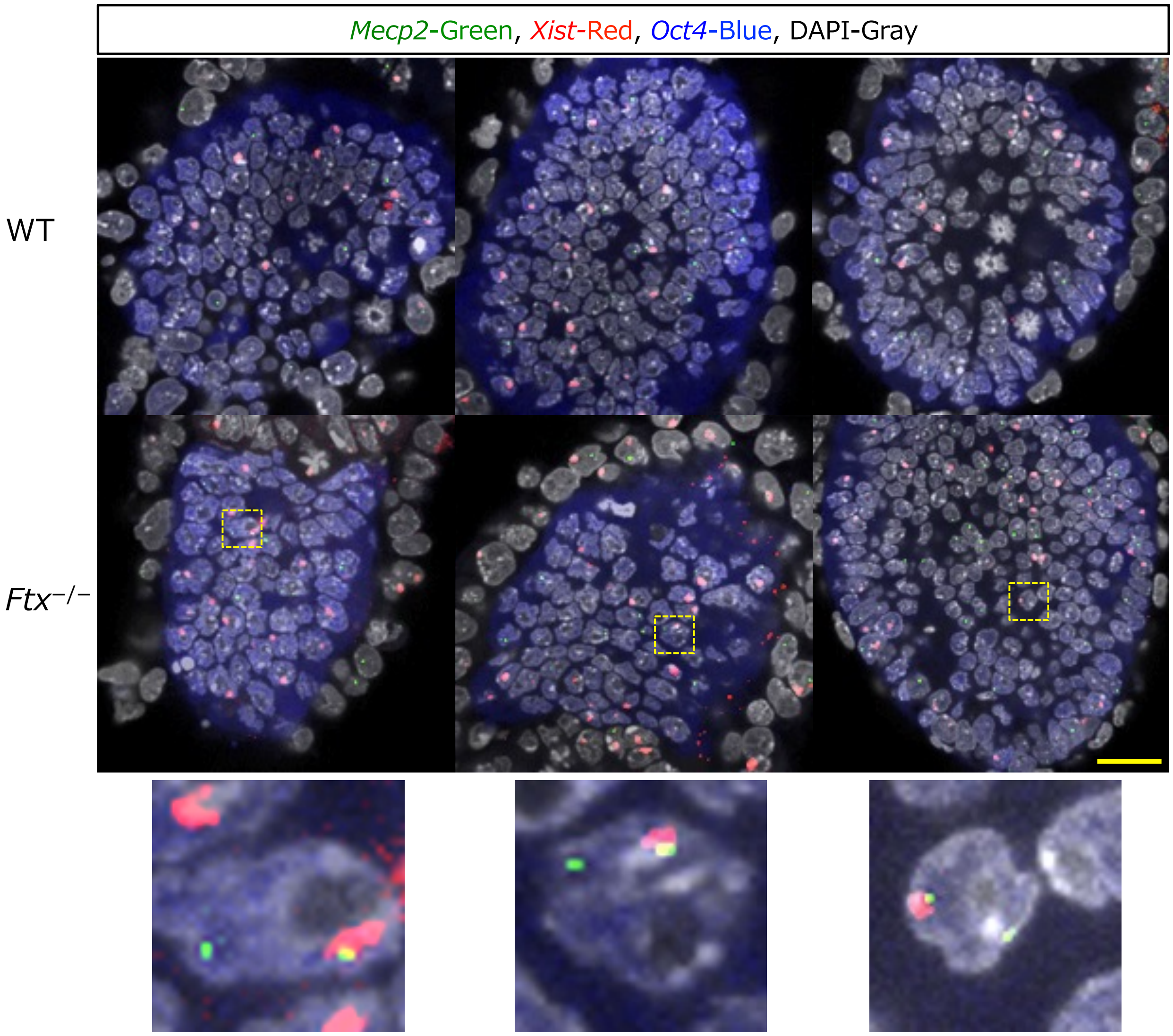

b

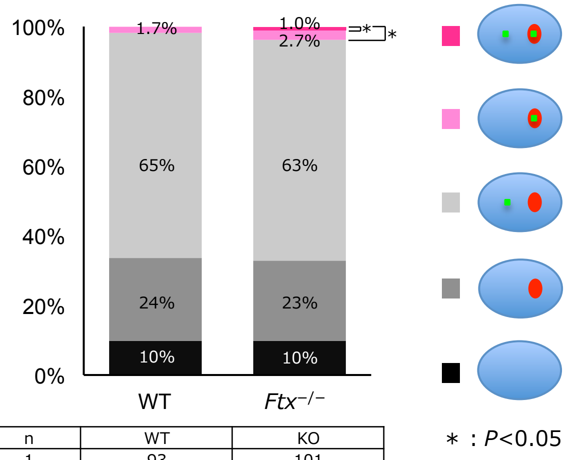

n	WT	KO
1	93	101
2	140	133
3	122	145
4	113	92
5	—	112
Total nuclei	468	583

Supplementary Figure 8. *Ftx*$^{-/-}$ mice showed partial failure of random XCI at E6.5.
(**a**) RNA–FISH analysis of WT and *Ftx*$^{-/-}$ female embryos at E6.5, using *Xist* (red) and *Mecp2* (green) as probes. The epiblast lineage was stained with an anti-Oct-3/4 antibody (blue). The yellow boxed areas are shown enlarged in the lower panels. Scale bar = 20 mm. (**b**) Frequency of ectopic *Mecp2* expression from the inactive X chromosome at E6.5. Dark and light pink indicate cells showing ectopic *Mecp2* expression from the inactive X chromosome judged as a signal within the *Xist* cloud (the percentage of the cells labelled in dark pink showed signals from active X and inactive X; the percentage of the cells labelled in light pink showed signals from inactive X only). Optical sections of whole embryos were obtained using confocal microscopy Z-stacks and were used to reconstruct three-dimensional images for counting the RNA–FISH signals. At least 90 nuclei were counted for each sample, and statistically significant differences were detected between WT and *Ftx*$^{-/-}$ embryos using Chi-squared tests (*P < .05).

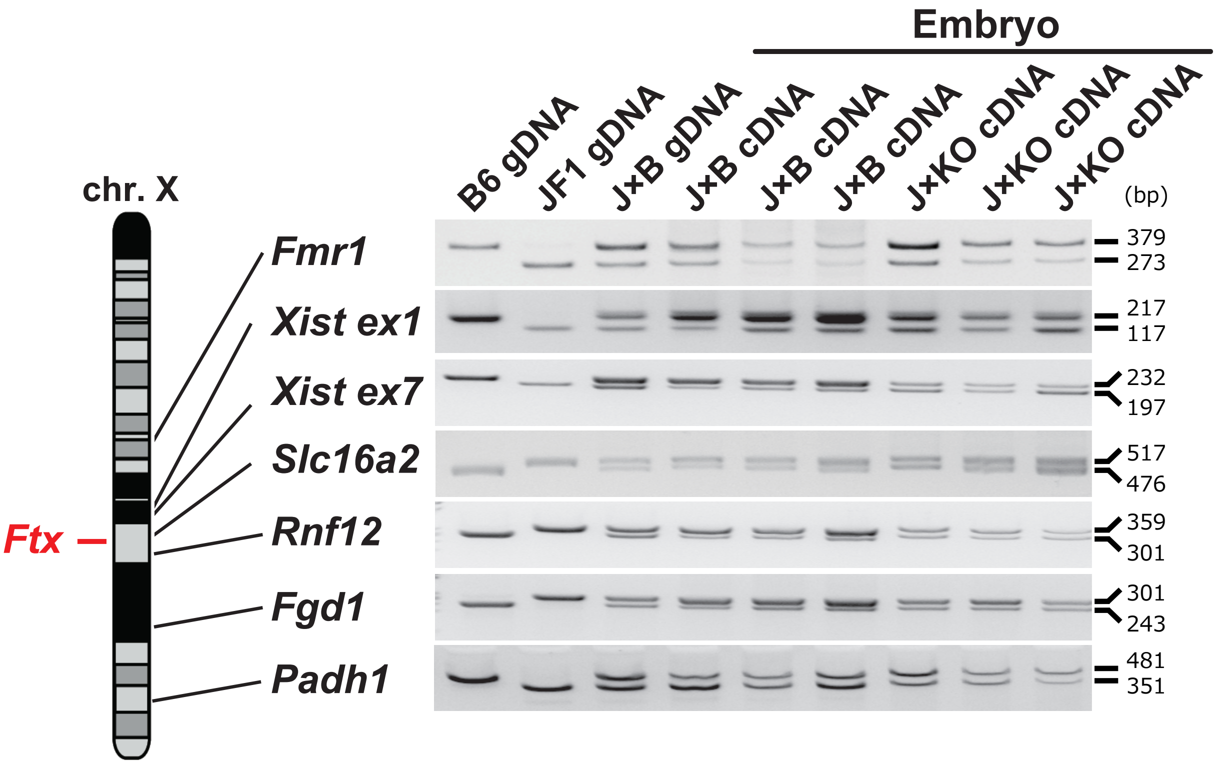

Supplementary Figure 9. Allelic expression analysis of X-linked genes in *Ftx*$^{+/-}$ postimplantation embryos (E9.5). Polymorphisms in X-linked genes between WT JF1 and *Ftx*-deficient B6 alleles were detected by RFLP analysis. The non-upregulated *Fmr1*, *Rnf12*, *Fgd1* and *Padh1* genes are known to be subjected to XCI. B6 gDNA, B6 genomic DNA; JF1 gDNA, JF1 genomic DNA; J × B gDNA, (JF1 × B6) F1 genomic DNA; J × B cDNA, JF1 × B6 RT–qPCR products; J × KO cDNA, JF1 × KO RT–qPCR products.

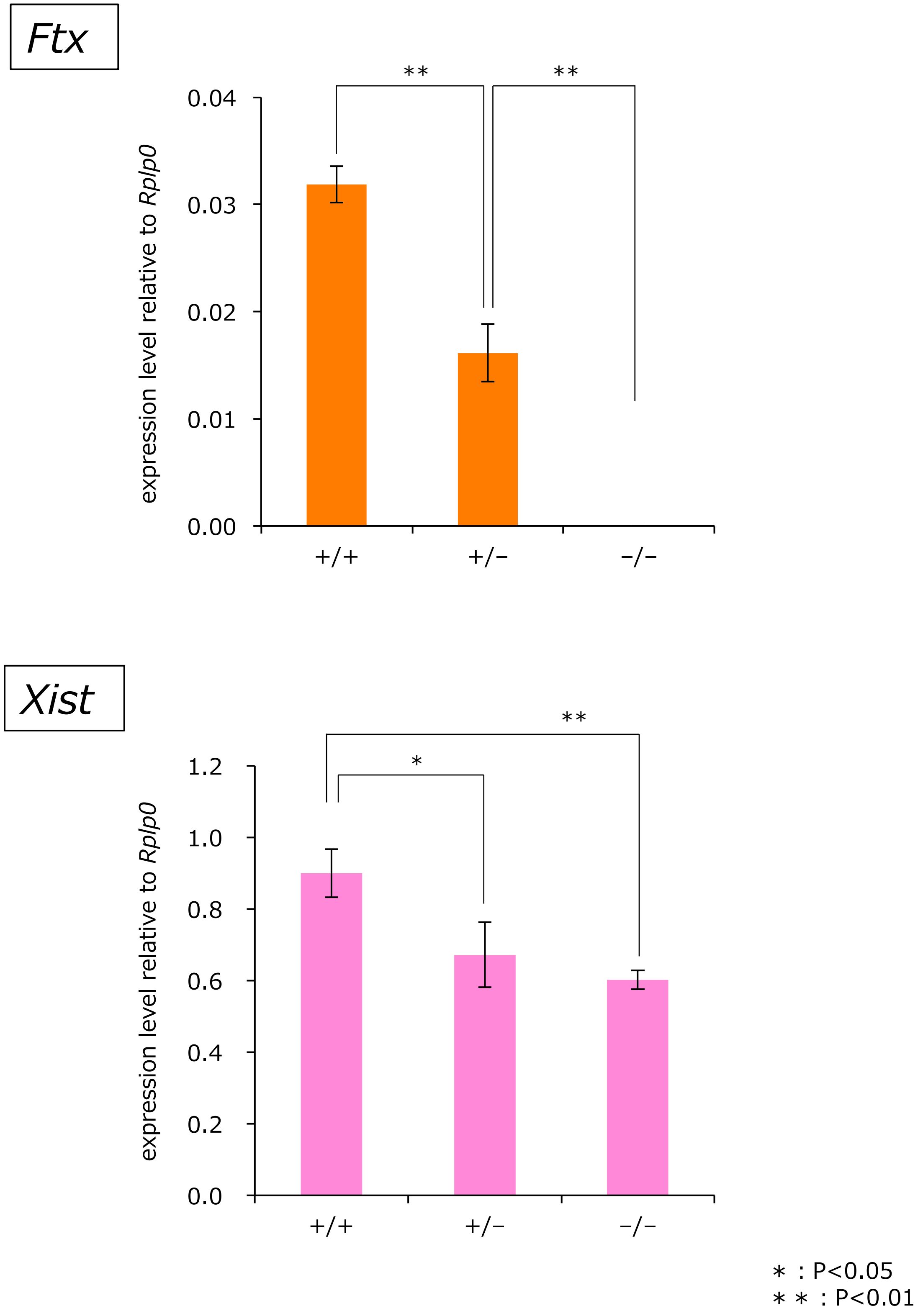

Supplementary Figure 10. RT–qPCR analysis of *Ftx* and *Xist* in WT females (+/+), heterozygous female (+/–) and homozygous female (–/–) eyes (*n* = 3, E13.5).
The PCR primers used in this analysis were as reported[7]. Significance levels of mean differences were calculated using a two-tailed, unequal variance *t*-test. Error bars, s.d. *Rplp0*: ribosomal protein, large at P0.

a

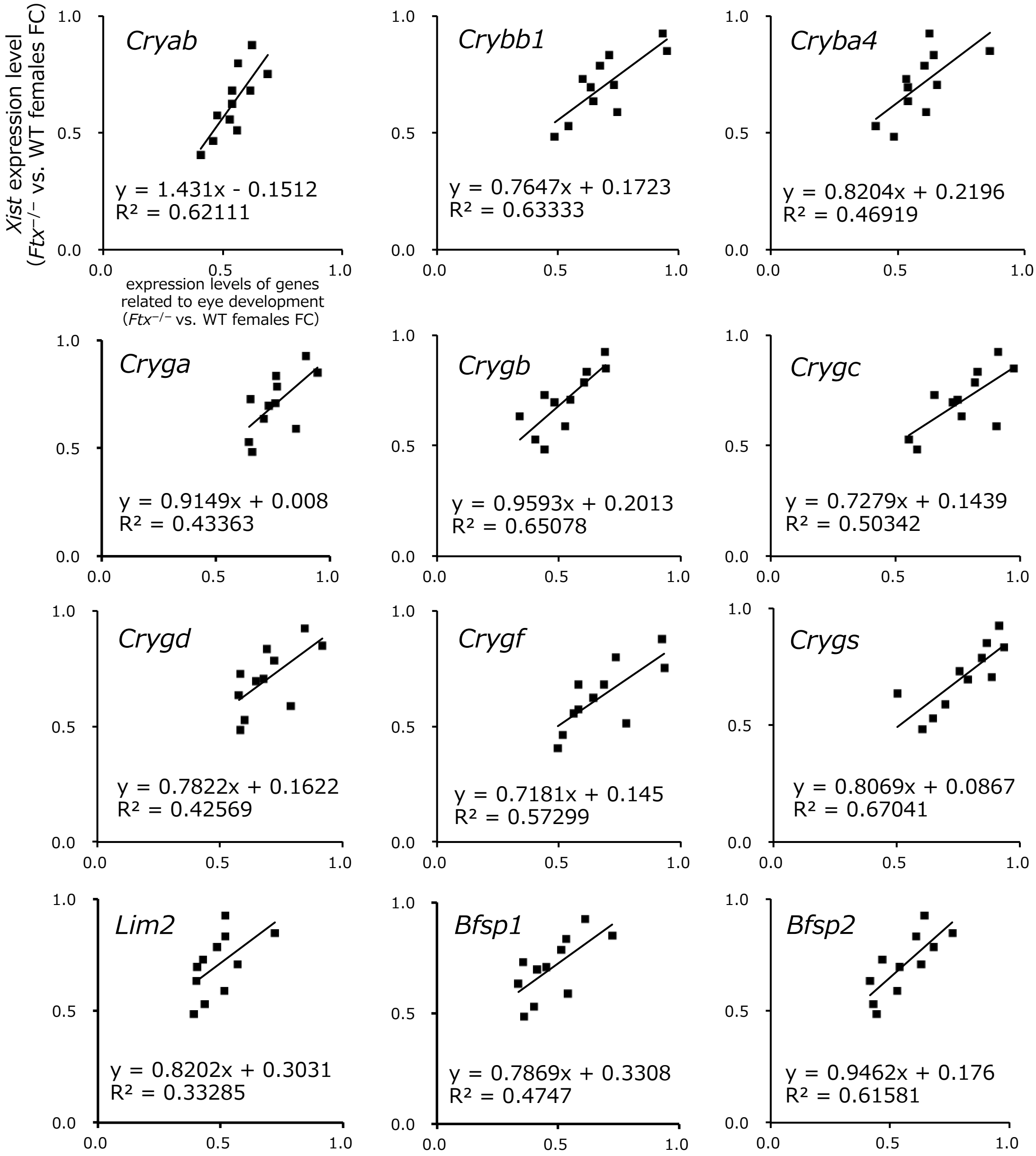

b

gene name	human phenotypes	mouse phenotypes	references (mutant of mice)
Cryab	cataract	microphthalmia, cataract	Andley UP, 2011 [1]
Cryba4	cataract	not determined	
Crybb1	cataract	not determined	
Cryga	not determined	microphthalmia, cataract	Graw J, 2004 [2]
Crygb	cataract	cataract	Li L, 2008 [3]
Crygc	microcornea, cataract	microphthalmia, cataract	Graw J, 2002 [4]
Crygd	microcornea, cataract	lens smaller, cataract	Graw J, 2002 [5]
Crygf	not determined	cataract	Graw J, 2002 [6]
Crygs	cataract	cataract	Lei Bu, 2002 [7]
Bfsp1	cataract	not determined	
Bfsp2	cataract	not determined	
Lim2	microphthalmia, cataract	microphthalmia, cataract	Steele EC Jr, 1997 [8] 2000 [9]

Human phenotypes refer to the Human–Mouse MGI database. (http://www.informatics.jax.org/humanDisease.shtml)

Supplementary Figure 11. Correlation between the expressions of *Xist* and autosomal genes with functions related to the Gene Ontogeny (GO) category "structural constituent of eye lens".
(**a**) In the eyes of female *Ftx*$^{-/-}$mice (n = 11), a correlation was found between the expression level of *Xist* and genes with functions related to this GO category. The Y axis shows *Xist* expression (*Ftx*$^{-/-}$ vs. WT females as FC values); the X axis shows the expression levels of each gene (*Ftx*$^{-/-}$ vs. WT females as FC values). (**b**) A list of the genes with functions related to structural constituents of the eye lens. Information on the phenotype of each mutant in mice and/or humans is also included.

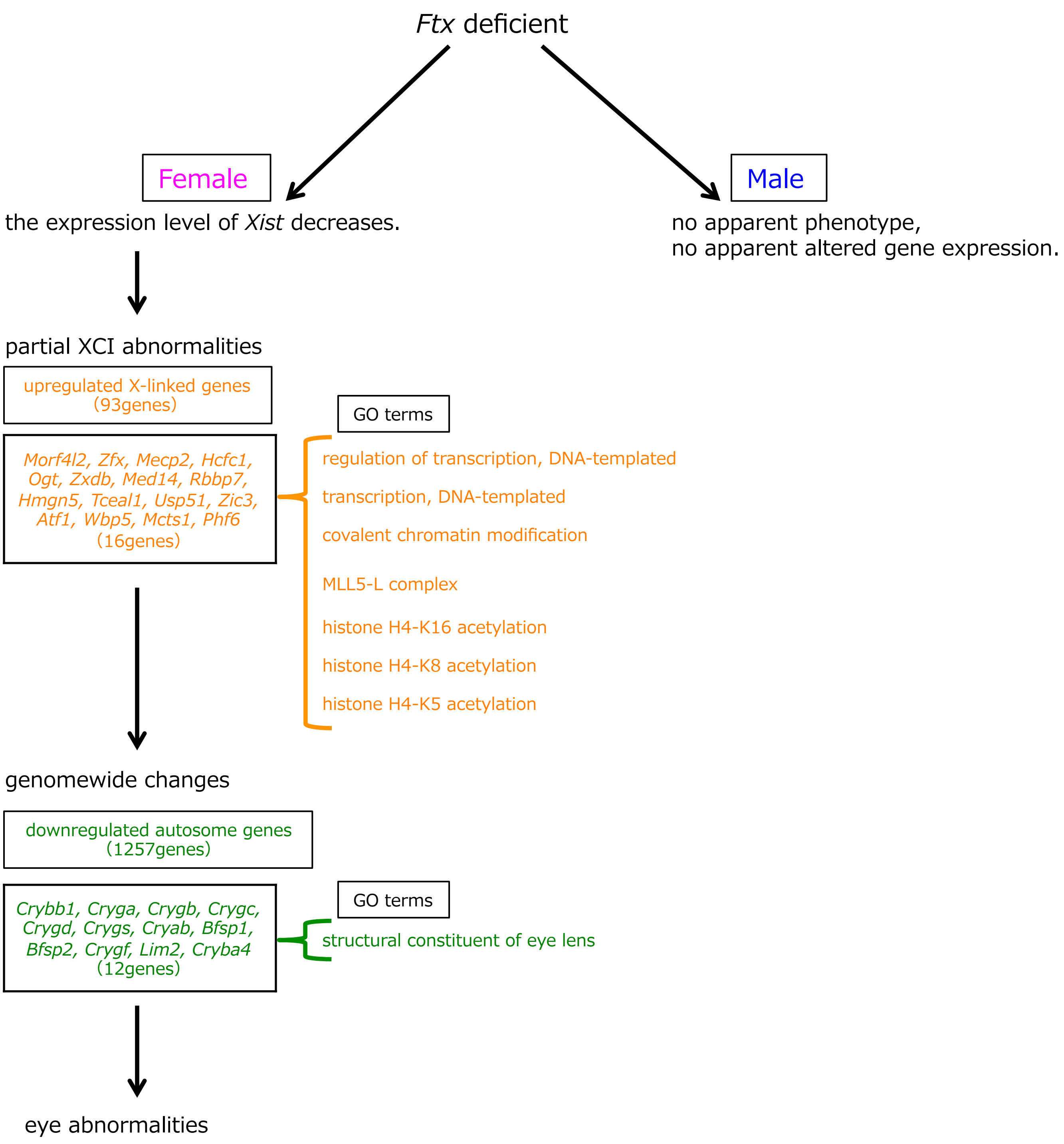

Supplementary Figure 12. A model for the eye abnormalities resulting from *Ftx* functional loss.
Ftx deficiency in female mice resulted in diminished expression of *Xist* and partial failure of XCI. GO analysis revealed that the upregulated X-linked genes showed significant enrichment of genetic pathways involved in the regulation of transcription (e.g., *Wbp5*, *Med14* and *Tceal1*) or in epigenetic control (e.g., *Hcfc1*, *Ogt* and *Mec*). Upregulated X-linked gene expression might result in alterations of the downstream expressions of autosomal genes. These autosomal alterations were barely detected in *Ftx*-deficient male mice (Ftx^{-}/Y), suggesting that *Ftx* does not act in *trans* to regulate autosomal genes directly. Altered gene expressions in $Ftx^{-/-}$ female mice involved some downregulated genes that are critical for normal eye development (e.g., *Crybb1*, *Ceyga* and *Bfsp1*). The expression levels of these eye-related genes were not changed in Ftx^{-}/Y male mice, suggesting that these eye abnormalities were caused by a partial failure of XCI.

Supplementary Table 1. Mating of *Ftx*-deficient mice (#64)

Cross	Male pups		Female pups		Total pups	Mean litter size
(♀×♂)	+/Y		+/-	+/0		
+/+ × -/Y	50		61	1		
	50		62		112	7.0

chi-square=0.30

Cross	Male pups		Female pups		Total pups	Mean litter size
(♀×♂)	+/Y	-/Y	+/+	-/+		
+/- × +/Y	53	42	48	37		
	95		85		180	7.0

chi-square=0.61

Cross	Male pups		Female pups		Total pups	Mean litter size
(♀×♂)	+/Y	-/Y	+/-	-/-		
+/- × -/Y	30	31	29	25		
	61		54		115	6.4

chi-square=0.87

Cross	Male pups	Female pups	Total pups	Mean litter size
(♀×♂)	-/Y	-/-		
-/- × -/Y	56	62	118	6.9

chi-square=0.58

Supplementary Table 2. Mating of *miRNA314/421* -deficient mice

Cross (♀×♂)	Male pups +/Y	Female pups +/-	Female pups +/0	Total pups	Mean litter size
+/+ × -/Y	16	24	1	41	8.2

Cross (♀×♂)	Male pups +/Y	Male pups -/Y	Female pups +/+	Female pups -/+	Total pups	Mean litter size
+/- × +/Y	32	33	35	33		
	65		68		133	7.8

Cross (♀×♂)	Male pups +/Y	Male pups -/Y	Female pups +/-	Female pups -/-	Total pups	Mean litter size
+/- × -/Y	22	16	12	15		
	38		27		65	8.3

Cross (♀×♂)	Male pups -/Y	Female pups -/-	Total pups	Mean litter size
-/- × -/Y	27	32	59	7.4

Supplementary Table 3.　Gene Ontology analysis for X-linked Up-regulation genes（P<0.05, Non-adjusted p-value）.

Category	Term	Count	%	PValue	Genes	List Total	Pop Hits	Pop Total	Fold Enrichment	Bonferroni	Benjamini	FDR
	GO:0006355~regulation of transcription, DNA-templated	15	22.058824	0.0022496	MORF4L2, ZFX, MECP2, HCFC1, Z	50	2279	18082	2.380	0.521184	0.521184	3.0030048
	GO:0006351~transcription, DNA-templated	13	19.117647	0.0037426	WBP5, MORF4L2, ZFX, MECP2, Z	50	1885	18082	2.494	0.7065751	0.4583129	4.9497626
	GO:0016569~covalent chromatin modification	5	7.3529412	0.0057723	MORF4L2, HCFC1, OGT, HMGN5,	50	266	18082	6.798	0.8493806	0.4679404	7.5381752
	GO:1901911~adenosine 5'-(hexahydrogen pentaphosphate) catabolic process	2	2.9411765	0.0107964	NUDT10, NUDT11	50	4	18082	180.820	0.9712646	0.5882778	13.667347
	GO:1901907~diadenosine pentaphosphate catabolic process	2	2.9411765	0.0107964	NUDT10, NUDT11	50	4	18082	180.820	0.9712646	0.5882778	13.667347
Biological Process	GO:0071543~diphosphoinositol polyphosphate metabolic process	2	2.9411765	0.0107964	NUDT10, NUDT11	50	4	18082	180.820	0.9712646	0.5882778	13.667347
	GO:1901909~diadenosine hexaphosphate catabolic process	2	2.9411765	0.0107964	NUDT10, NUDT11	50	4	18082	180.820	0.9712646	0.5882778	13.667347
	GO:0006357~regulation of transcription from RNA polymerase II promoter	5	7.3529412	0.0223119	WBP5, MED14, HMGN5, TCEAL1,	50	397	18082	4.555	0.9993755	0.7713874	26.323888
	GO:0043984~histone H4-K16 acetylation	2	2.9411765	0.0372904	HCFC1, OGT	50	14	18082	51.663	0.999996	0.8739641	40.220688
	GO:0043982~histone H4-K8 acetylation	2	2.9411765	0.0425051	HCFC1, OGT	50	16	18082	45.205	0.9999993	0.8685373	44.458774
	GO:0043981~histone H4-K5 acetylation	2	2.9411765	0.0425051	HCFC1, OGT	50	16	18082	45.205	0.9999993	0.8685373	44.458774
	GO:0005634~nucleus	31	45.588235	6.29E-04	MORF4L2, POLA1, VBP1, HCFC1,	59	6019	19662	1.716	0.0674371	0.0674371	0.7093149
	GO:0005654~nucleoplasm	13	19.117647	0.0098167	CSTF2, SYAP1, MORF4L2, POLA1,	59	1935	19662	2.239	0.6654743	0.4216181	10.563915
Cellular Component	GO:0005884~actin filament	3	4.4117647	0.0171989	AMOT, PLS3, GDPD2	59	68	19662	14.702	0.8542238	0.4737054	17.826284
	GO:0070688~MLL5-L complex	2	2.9411765	0.0262428	HCFC1, OGT	59	9	19662	74.056	0.9477575	0.5219141	25.989221
	GO:0005730~nucleolus	7	10.294118	0.0371223	MORF4L2, POLA1, HAUS7, HMGN	59	842	19662	2.771	0.9849895	0.5682033	34.826225
	GO:0052843~inositol-1-diphosphate-2,3,4,5,6-pentakisphosphate diphosphatase activity	2	2.9411765	0.0111884	NUDT10, NUDT11	50	4	17446	174.460	0.757725	0.757725	12.216713
	GO:0052847~inositol-1,5-bisdiphosphate-2,3,4,6-tetrakisphosphate 5-diphosphatase activity	2	2.9411765	0.0111884	NUDT10, NUDT11	50	4	17446	174.460	0.757725	0.757725	12.216713
	GO:0000298~endopolyphosphatase activity	2	2.9411765	0.0111884	NUDT10, NUDT11	50	4	17446	174.460	0.757725	0.757725	12.216713
	GO:0052848~inositol-3,5-bisdiphosphate-2,3,4,6-tetrakisphosphate 5-diphosphatase activity	2	2.9411765	0.0111884	NUDT10, NUDT11	50	4	17446	174.460	0.757725	0.757725	12.216713
	GO:0052840~inositol diphosphate tetrakisphosphate diphosphatase activity	2	2.9411765	0.0111884	NUDT10, NUDT11	50	4	17446	174.460	0.757725	0.757725	12.216713
	GO:0052846~inositol-1,5-bisdiphosphate-2,3,4,6-tetrakisphosphate 1-diphosphatase activity	2	2.9411765	0.0111884	NUDT10, NUDT11	50	4	17446	174.460	0.757725	0.757725	12.216713
	GO:0052844~inositol-3-diphosphate-1,2,4,5,6-pentakisphosphate diphosphatase activity	2	2.9411765	0.0111884	NUDT10, NUDT11	50	4	17446	174.460	0.757725	0.757725	12.216713
Molecular Function	GO:0008486~diphosphoinositol-polyphosphate diphosphatase activity	2	2.9411765	0.0111884	NUDT10, NUDT11	50	4	17446	174.460	0.757725	0.757725	12.216713
	GO:0034431~bis(5'-adenosyl)-hexaphosphatase activity	2	2.9411765	0.0111884	NUDT10, NUDT11	50	4	17446	174.460	0.757725	0.757725	12.216713
	GO:0052845~inositol-5-diphosphate-1,2,3,4,6-pentakisphosphate diphosphatase activity	2	2.9411765	0.0111884	NUDT10, NUDT11	50	4	17446	174.460	0.757725	0.757725	12.216713
	GO:0034432~bis(5'-adenosyl)-pentaphosphatase activity	2	2.9411765	0.0111884	NUDT10, NUDT11	50	4	17446	174.460	0.757725	0.757725	12.216713
	GO:0043996~histone acetyltransferase activity (H4-K8 specific)	2	2.9411765	0.0277414	HCFC1, OGT	50	10	17446	69.784	0.971126	0.8300766	27.805316
	GO:0046972~histone acetyltransferase activity (H4-K16 specific)	2	2.9411765	0.0277414	HCFC1, OGT	50	10	17446	69.784	0.971126	0.8300766	27.805316
	GO:0043995~histone acetyltransferase activity (H4-K5 specific)	2	2.9411765	0.0277414	HCFC1, OGT	50	10	17446	69.784	0.971126	0.8300766	27.805316
	GO:0050072~m7G(5')pppN diphosphatase activity	2	2.9411765	0.0304737	NUDT10, NUDT11	50	11	17446	63.440	0.9797461	0.7274145	30.120261
	GO:0003682~chromatin binding	5	7.3529412	0.0414343	MECP2, POLA1, HCFC1, HMGN5,	50	466	17446	3.744	0.9951654	0.7363124	38.741053

Supplementary Table 4. Gene Ontology analysis for Autosome Up-regulation genes（P<0.01, Bonferroni adjusted p-value）.

Category	Term	Count	%	PValue	Genes	List Total	Pop Hits	Pop Total	Fold Enrichment	Bonferroni	Benjamini	FDR
	GO:0007049~cell cycle	85	6.6510172	1.75E-12	ITGB3BP, SPIN1, E2F3, LZTS2, CDC	1100	614	18082	2.276	6.42E-09	6.42E-09	3.22E-09
	GO:0006355~regulation of transcription, DNA-templated	208	16.27543	7.68E-10	ITGB3BP, MEF2A, SNIP1, RORB, RE	1100	2279	18082	1.500	2.82E-06	1.41E-06	1.41E-06
	GO:0006397~mRNA processing	50	3.9123631	2.50E-09	SRSF1, SRSF10, STRAP, PNPT1, SN	1100	322	18082	2.553	9.16E-06	3.05E-06	4.60E-06
	GO:0006351~transcription, DNA-templated	176	13.771518	4.32E-09	MORF4L1, ITGB3BP, MEF2A, ARNT	1100	1885	18082	1.535	1.58E-05	3.96E-06	7.95E-06
Biological Process	GO:0008380~RNA splicing	40	3.1298905	1.93E-08	SRSF1, SRSF10, STRAP, SNRPB2, C	1100	241	18082	2.728	7.09E-05	1.42E-05	3.56E-05
	GO:0007067~mitotic nuclear division	43	3.3646322	3.81E-08	HAUS4, ITGB3BP, LZTS2, HAUS1, C	1100	277	18082	2.552	1.40E-04	2.33E-05	7.01E-05
	GO:0051301~cell division	52	4.0688576	4.95E-08	ITGB3BP, LZTS2, CDC14A, AURKA,	1100	374	18082	2.286	1.82E-04	2.59E-05	9.12E-05
	GO:0051028~mRNA transport	21	1.6431925	2.33E-07	SRSF1, XPO1, MYO1C, KIF5C, HNR	1100	88	18082	3.923	8.56E-04	1.07E-04	4.30E-04
	GO:0000122~negative regulation of transcription from RNA polymerase	78	6.1032864	1.37E-06	BMI1, XPO1, MEF2A, HMGN2, E2F7,	1100	729	18082	1.759	0.0050258	5.60E-04	2.53E-03
	GO:0005634~nucleus	584	45.696401	8.39E-48	RPL17, MEF2A, PPP2R5C, RORB, RE	1158	6019	19662	1.647	5.88E-45	5.88E-45	1.27E-44
	GO:0005737~cytoplasm	556	43.505477	1.98E-25	MEF2A, RPL14, CLK1, ACBD6, CTN	1158	6631	19662	1.424	1.39E-22	6.94E-23	2.99E-22
	GO:0005654~nucleoplasm	224	17.527387	4.20E-24	ITGB3BP, RAD51C, MEF2A, SNIP1, [illegible]	1158	1935	19662	1.966	2.94E-21	9.79E-22	6.33E-21
	GO:0005730~nucleolus	109	8.5289515	1.00E-14	XPO1, SRP19, SETX, ESF1, CUL2, W	1158	842	19662	2.198	6.99E-12	1.75E-12	1.51E-11
	GO:0005829~cytosol	166	12.989045	2.04E-09	TBK1, AP1G1, VPS54, BTRC, FOXO1	1158	1784	19662	1.580	1.43E-06	2.85E-07	3.07E-06
	GO:0043231~intracellular membrane-bounded organelle	80	6.2597809	3.51E-07	PTGES3, XPO1, GPBP1, AP1G1, FOX	1158	751	19662	1.809	2.46E-04	4.10E-05	5.29E-04
Cellular Component	GO:0030529~intracellular ribonucleoprotein complex	44	3.4428795	3.62E-07	MYO5A, XPO1, RPL17, RPL14, SNR	1158	320	19662	2.335	2.53E-04	3.62E-05	5.45E-04
	GO:0005925~focal adhesion	50	3.9123631	4.80E-07	KIF22, GJA1, CLTC, IQGAP1, CTNN	1158	391	19662	2.171	3.36E-04	4.20E-05	7.23E-04
	GO:0070062~extracellular exosome	212	16.588419	2.98E-06	RPL14, ATP1B3, LUZP1, CCT2, B2M	1158	2674	19662	1.346	0.0020833	2.32E-04	0.0044929
	GO:0005913~cell-cell adherens junction	41	3.2081377	4.14E-06	PARD3, CNN3, STK38, RPL14, SNX	1158	316	19662	2.203	0.0028972	2.90E-04	0.0062506
	GO:0005681~spliceosomal complex	24	1.8779343	4.30E-06	SRSF1, SREK1, SNRPB2, CWC15, H	1158	136	19662	2.996	0.0030074	2.74E-04	0.0064887
	GO:0000932~cytoplasmic mRNA processing body	17	1.3302034	4.92E-06	POLR2G, MEX3B, CNOT2, CNOT1, W	1158	74	19662	3.901	0.0034375	2.87E-04	0.0074182
	GO:0005694~chromosome	43	3.3646322	6.06E-06	ITGB3BP, HMGB1, HMGB2, PPP2R5	1158	344	19662	2.122	0.0042304	3.26E-04	0.0091327
	GO:0005938~cell cortex	24	1.8779343	1.15E-05	PARD3, MAP2K1, FRYL, ARHGEF7, [illegible]	1158	144	19662	2.830	0.007993	5.73E-04	0.0172876
	GO:0044822~poly(A) RNA binding	162	12.676056	4.37E-26	RPL17, RPL14, SNIP1, RBM5, CNOT	1064	1113	17446	2.387	4.69E-23	4.69E-23	6.97E-23
	GO:0005515~protein binding	368	28.794992	9.97E-18	HMGN1, RAD51C, RPL17, MEF2A, A	1064	4092	17446	1.475	1.07E-14	5.34E-15	1.59E-14
	GO:0003723~RNA binding	98	7.6682316	1.05E-11	XPO1, RBM5, SRP19, TARDBP, ZRS	1064	780	17446	2.060	1.13E-08	3.75E-09	1.67E-08
	GO:0003677~DNA binding	169	13.223787	4.44E-08	HMGN1, RAD51C, MEF2A, HMGN2,	1064	1847	17446	1.500	4.76E-05	1.19E-05	7.07E-05
	GO:0061630~ubiquitin protein ligase activity	34	2.6604069	1.38E-07	BTRC, UBE2V2, TRIM71, TRIM11, R	1064	199	17446	2.801	1.47E-04	2.95E-05	2.19E-04
Molecular Function	GO:0003682~chromatin binding	58	4.5383412	3.90E-07	MORF4L1, HMGN1, BMI1, MEF2A, M	1064	466	17446	2.041	4.18E-04	6.97E-05	6.22E-04
	GO:0003676~nucleic acid binding	119	9.3114241	5.70E-07	TBK1, RBM5, REST, ZFP84, ZFP932	1064	1237	17446	1.577	6.11E-04	8.73E-05	9.09E-04
	GO:0046872~metal ion binding	267	20.892019	9.70E-07	PDP1, RORB, EPT1, REST, ITSN1, Z	1064	3355	17446	1.305	1.04E-03	1.30E-04	1.55E-03
	GO:0004842~ubiquitin-protein transferase activity	44	3.4428795	1.45E-06	HECW1, BTRC, FEM1B, TRIM71, TT	1064	326	17446	2.213	0.0015584	1.73E-04	0.002319
	GO:0008134~transcription factor binding	45	3.5211268	2.16E-06	HMGB2, E2F5, SOX2, FOXO1, NFYC,	1064	342	17446	2.157	0.0023086	2.31E-04	0.0034366
	GO:0098641~cadherin binding involved in cell-cell adhesion	39	3.0516432	2.72E-06	CNN3, STK38, RPL14, SNX5, CAPZA	1064	279	17446	2.292	0.0029116	2.65E-04	0.0043355
	GO:0000166~nucleotide binding	165	12.910798	5.74E-06	RAD51C, STK38, TBK1, RASL2-9, R	1064	1936	17446	1.397	0.0061321	5.12E-04	0.0091455

Supplementary Table 5.   Gene Ontology analysis for Autosome Down-regulation genes（P<0.01, Bonferroni adjusted p-value）.

Category	Term	Count	%	PValue	Genes	List Total	Pop Hits	Pop Total	Fold Enrichment	Bonferroni	Benjamini	FDR
Biological Process	GO:0051290~protein heterotetramerization	12	1.2698413	3.20E-06	HIST4H4, HIST1H4K, HIST1H3C, H	768	47	18082	6.011	9.51E-03	9.51E-03	5.76E-03
Cellular Component	GO:0005634~nucleus	364	38.518519	1.62E-16	SCAF1, S100A4, S100A6, PDLIM7,	827	6019	19662	1.438	6.53E-14	6.53E-14	1.67E-13
	GO:0005737~cytoplasm	364	38.518519	2.88E-10	SYT1, RAB3GAP2, S100A6, PDLIM7	827	6631	19662	1.305	1.70E-07	8.48E-08	4.25E-07
	GO:0005829~cytosol	118	12.486772	7.18E-07	EIF6, S100A6, STAT5B, PGAM2, HC	827	1784	19662	1.573	4.22E-04	1.41E-04	0.0010577
	GO:0070062~extracellular exosome	160	16.931217	2.39E-06	EIF6, S100A4, TSPO, S100A6, ALDH	827	2674	19662	1.423	0.0014072	3.52E-04	0.0035271
	GO:0005913~cell-cell adherens junction	33	3.4920635	4.19E-06	SNX9, YWHAZ, CADM3, PARD3, HD	827	316	19662	2.483	0.0024613	4.93E-04	0.0061725
Molecular Function	GO:0005212~structural constituent of eye lens	12	1.2698413	1.38E-09	CRYBB1, CRYGA, CRYGB, CRYGC, C	756	24	17446	11.538	1.22E-06	1.22E-06	2.15E-06
	GO:0005515~protein binding	244	25.820106	1.40E-08	S100A4, SYT1, S100A6, CADM3, PD	756	4092	17446	1.376	1.24E-05	6.21E-06	2.18E-05

Supplementary Table 6. A list of the PCR primers used in this research.

	gene name	primer name		sequnece (5' →3')
Construction of *miRNA374/421* disruption vector	*Fas2* -L-F1+SpeI	Forward	003	aaactagtagcaatcctcagaccaacga
	Fas2 -L-F2+SpeI	Forward	004	aaactagtcctaacatggatgtcgcaga
	Fas2 -L-F3	Forward	005	aaattccatgcataagagatttataaa
	Fas2 -L-F4	Forward	006	taaatgtaaccaagatctcagaaatgaa
	Fas2 -M-F2-As	Forward	008	aaggcgcgccagctgcagaaataaggacgctg
	Fas2 gF1	Forward	057	ttctggagctatgagcagca
	Fas2 gR1	Reverse	058	caacctggagtggtgttcct
	Fas2 gF4	Forward	059	tccagggatgacagtcacaa
	Fas2 gR5	Reverse	060	cacagttgacctgaagccct
	Fas2 gR6	Reverse	061	cccatgtaggaaactcgatgattc

	gene name	primer name		sequnece (5' →3')
Validation of recombination	*miRNA374/421* short arm	Forward	001	gccttctatcgccttcttgacgagttcttc
		Reverse	024	agaaaacaagcgcaggaactct
	miRNA374/421 long arm	Forward	041	gagtagaaggtggcgcgaag
		Reverse	042	cactagacactgcccaacactaaac

	gene name	primer name		sequnece (5' →3')
Genotyping	*miRNA374/421*	Forward	330	ccgcgcaccatgagcacaac
		Forward	057	ttctggagctatgagcagca
		Reverse	061	cccatgtaggaaactcgatgattc

	gene name	primer name		sequnece (5' →3')	Restriction enzyme
polymorphic analysis(RFLP)	*Xist exon1*	Forward	105	ctaaaactcagcccgttcca	SpeI
		Reverse	106	gcaaccccagcaatagtcat	
	Xist exon 7	Forward	107	gcccaggtcacattatggtt	SacI
		Reverse	108	ctccaatttctgggctcaag	
	Ftx	Forward	116	gccatctgatgtcatgttgg	HpyCH4Ⅲ
		Reverse	117	ggtgttggttctcttgctcc	
	Rnf12	Forward	094	ctggagagtcttcagatgatgtga	HaeⅢ
		Reverse	091	ggtcggcacttctgttactgc	
	Fmr1	Forward	079	cttaacacttcagggcagg	RsaI
		Reverse	080	cttccctgaactctgcatcc	
	Fgd1	Forward	084	tcacacaaagccacctaagc	HhaI
		Reverse	085	attgactgcattgggagtgg	
	Slc16a2	Forward	109	tgtacggctcacctcattagg	BseRI
		Reverse	110	ggaagtggaagcattgttgc	
	Padh1	Forward	086	ttccagcgatatgctgacttt	TaqI
		Reverse	087	tggcaaggcatgaagtgata	
	Mecp2	Forward	800	cccatctcctcatgattgct	StuI
		Reverse	794	ggccgtgctagcaaagtaag	
	Ogt	Forward	795	ttatgcagctggcaacaaac	ScaI
		Reverse	799	tcccctccacattaagcatc	

Supplementary Table 7. A list of the qPCR primers and probes used in this research.

	gene name	primer name	sequnece (5' →3')
qPCR	*beta-actin*	Forward 051	aagtgtgacgttgacatccg
		Reverse 052	gatccacatctgctggaagg
	Ftx	Forward 207	atcttcttgcgctcctcctt
		Reverse 208	tgtgtccagggctgtctgt
	Xist	Forward 236	cagagtagcgaggacttgaagag
		Reverse 237	gctggttcgtctatcttgtggg

	gene name	Assay Name	Assay ID
qPCR_TaqMan MicroRNA Assay （ABI）	*miRNA374*	mmu-miR-374-5p	001319
	miRNA421	mmu-miR-421-3p	002700
	miRNA295	mmu-miR-295	000189

	gene name	Assay ID
qPCR_TaqMan Gene Expression Assay（ABI）	*Xist*	Mm01232884_m1
	B2m	Mm00437762_m1

	gene name	primer + probe	sequnece (5' →3')
qPCR_PrimeTime qPCR Assay（IDT）	*Tmem29*	Forward	GACCAGGACATACAGGAACATC (Sense)
		Probe	AGGAACCAAGGCAGCATCAATAACCC (Sense)
		Reverse	CTGCTTTCTGGTACTCTCTCAC (AntiSense)
	Mecp2	Forward	GGTTGTCTCCACTGCTACTTAC (Sense)
		Probe	CATGGTCACCTACCTGTCAGTGGC (AntiSense)
		Reverse	GCTAACTTGGGTGCTGATCT (AntiSense)
	Ogt	Forward	ACAGAGGGCAGATTCAGATAAC (Sense)
		Probe	AGCAATGGACTGGCGACTACACAG (Sense)
		Reverse	CTCCGGTTGCAGCCTTATTA (AntiSense)
	Gab3	Forward	CAAGACCACTTCCCGTACATTC (Sense)
		Probe	TGAGGAAGAAATGCAGGTGTGGGT (Sense)
		Reverse	CCATGGAATCTGCACCATCTT (AntiSense)
	Slc16a2	Forward	TCCATCCCTGGACTTAAGAAGA (Sense)
		Probe	CTCATTCCTGCTCCTGGGCTTGAT (Sense)
		Reverse	GGAAGAGGCAGACAACGATAAG (AntiSense)

Supplemental references

1. Andley, U.P., Hamilton, P.D., Ravi, N. & Weihl, C.C. A knock-in mouse model for the R120G mutation of αB-crystallin recapitulates human hereditary myopathy and cataracts. *PLoS One* **6,** e17671 (2011).
2. Graw, J. *et al.* Genetic and allelic heterogeneity of *Cryg* mutations in eight distinct forms of dominant cataract in the mouse. *Invest. Ophthalmol. Vis. Sci.* **45,** 1202-1213 (2004).
3. Li, L. *et al.* Dense nuclear cataract caused by the γB-Crystallin S11R point mutation. *Invest. Ophthalmol. Vis. Sci.* **49,** 304–309 (2008).
4. Graw, J., Neuhauser-Klaus, A., Loster, J. & Favor, J. A 6-bp deletion in the Crygc gene leading to a nuclear and radial cataract in the mouse. *Invest. Ophthalmol. Vis. Sci.* **43,** 236–240 (2002).
5. Graw, J. *et al.* V76D mutation in a conserved gD-crystallin region leads to dominant cataracts in mice. *Mamm. Genome* **13,** 452–455 (2002).
6. Graw, J., Klopp, N., Nehauser-Klaus, A., Favor, J. & Löster, J. *Crygf* ^{Rop}: The first mutation in the *Crygf* gene causing a unique radial lens opacity. *Invest. Ophthalmol. Vis. Sci.* **43,** 2998–3002 (2002).
7. Bu, L. *et al.* The γS-crystallin gene is mutated in autosomal recessive cataract in mouse. *Genomics* **80,** 38–44 (2002).
8. Steele, E.C., Jr. *et al.* Identification of a mutation in the MP19 gene, *Lim2*, in the cataractous mouse mutant *To3*. *Mol. Vis.* **3,** 9238094 (1997).
9. Steele, E.C., Jr. *et al.* *Lim2*^{To3} transgenic mice establish a causative relationship between the mutation identified in the *lim2* gene and cataractogenesis in the *To3* mouse mutant. *Mol. Vis.* **6,** 85–94 (2000).
