# Supplementary material for: Female mice lacking Ftx lncRNA exhibit impaired X-chromosome inactivation and a microphthalmia-like phenotype
Source: Nat Commun. 2018 Sep 20;9:3829. doi: 10.1038/s41467-018-06327-6 (PMC6148026; doi:10.1038/s41467-018-06327-6)
Supplement: Supplementary file 3 — Description of Additional Supplementary Files [file 41467_2018_6327_MOESM3_ESM.pdf]

Description of Additional Supplementary Files:

Supplementary Data 1: Microarray data of X-linked genes.

Supplementary Data 2: Microarray data of Autosomal genes.
